# Perioperative Considerations for a Patient with a Left Ventricular Assist Device Undergoing Thyroidectomy

**DOI:** 10.7759/cureus.7132

**Published:** 2020-02-28

**Authors:** Avijit Sharma, Paula Trigo-Blanco, Adriana D Oprea

**Affiliations:** 1 Anesthesiology, Yale School of Medicine, New Haven, USA; 2 Anesthesiology, Southern New Hampshire Medical Center, Nashua, USA

**Keywords:** left ventricular assist device, thyroidectomy

## Abstract

As the population ages, ventricular assist devices (VADs) are becoming more prevalent, even in the outpatient perioperative setting. Patients with VADs present unique challenges for the anesthesiologist, who needs to have a thorough understanding of device physiology and design an appropriate anesthetic plan. This case report demonstrates an alternative monitored anesthesia care (MAC) anesthetic technique for a patient with a left VAD undergoing total thyroidectomy. This alternative provided a safe comfortable anesthetic and can be used successfully in patients with circulatory support devices.

## Introduction

As the population ages, the number of patients with left ventricular assist devices (LVADs) has drastically increased. One in five Americans over the age of 40 develops heart failure and many of these patients require cardiac support devices [1]. VADs are most commonly utilized as destination therapy, as a bridge to candidacy, or as a bridge to transplantation [1]. With more devices being implanted, it is imperative to understand the physiology and management of patients supported by these devices when they arrive at the operating room. The patient we are presenting had severe cardiomyopathy, thus requiring an LVAD. We safely administered anesthesia for his total thyroidectomy by using a superficial cervical block and monitored anesthesia care (MAC). Consent to report this case was obtained from the patient.

## Case presentation

A 68-year-old male presented for total thyroidectomy due to amiodarone-induced thyrotoxicosis. Past medical history included ischemic cardiomyopathy (left ventricular ejection fraction 37%) supported by a Heartmate 3 LVAD (Abbott Laboratories, Lake Bluff, Illinois) biventricular implantable cardioverter-defibrillator (BiV-ICD) placement, atrial fibrillation on chronic amiodarone, hypertension, and obesity. The patient had presented to the hospital a month prior, in cardiogenic shock, with a further decrease of his ejection fraction to 21%. He required an intra-aortic balloon pump and subsequent placement of a Heartmate 3 LVAD. The hospital course was further complicated by a tenuous volume status requiring alternating diuretic dosing with pressor/albumin support. More importantly, the patient was diagnosed with amiodarone-induced thyrotoxicosis. Despite maximal medical treatment (methimazole, high dose prednisone, and cholestyramine), thyroid-stimulating hormone (TSH) levels remained <0.005 (0.27-4.2 µUI/ml). Total thyroidectomy was recommended by a multidisciplinary care team (endocrinologist, endocrine surgeon, and cardiologist).

Preoperative transthoracic echocardiography showed LVAD present with mildly decreased right ventricular function. The electrocardiogram demonstrated sinus rhythm with premature ventricular complexes and a left bundle branch block.

**Figure 1:**
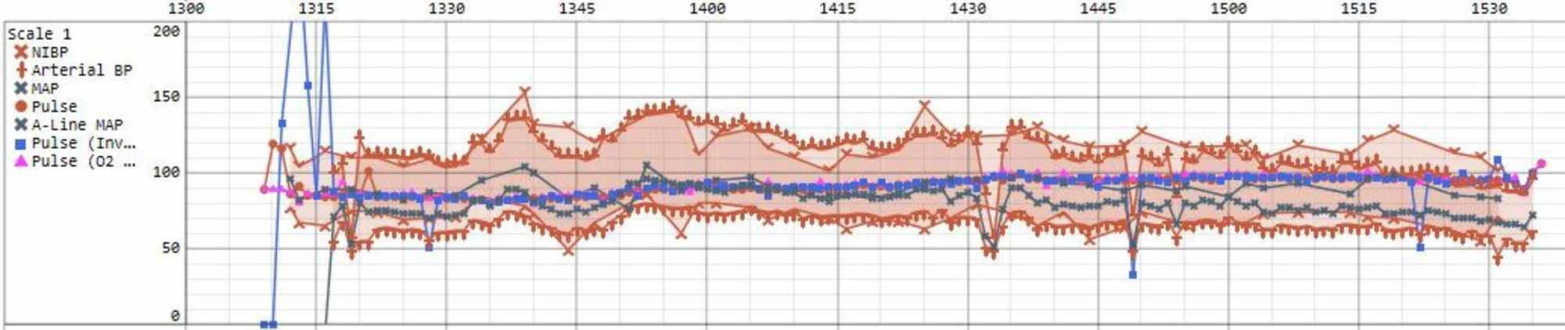
Intraoperative vital signs

Due to concerns about the patient’s cardiac status, it was decided to perform the surgery under MAC/superficial cervical block, with general anesthesia as a back-up option. The patient was brought to the operating room and his LVAD was connected to the operating room power source. An arterial line was placed for constant blood pressure measurements in addition to standard American Society of Anesthesiologists (ASA) monitoring. Per discussion with the surgical team, a bipolar cautery was used for the procedure so as not to interfere with the ICD function. The patient was given 2 mg of midazolam and an alfentanil infusion was started at 1.2 mcg/kg/min. He was additionally given 500 mcg bolus of alfentanil prior to the cervical block performed by the surgical team. Intravenous (IV) fluid was infused at 50 ml/hour and there was no vasopressor or blood transfusion requirement during the case. The patient tolerated the procedure extremely well from a hemodynamic standpoint (Figure 1) as well as the pain, comfort, and awareness standpoint. No conversion to general anesthesia or advanced airway was necessary for the case.

After completion of the surgery, he recovered in the intensive care unit in stable condition. There were no reported complications at postoperative follow-up.

## Discussion

Patients presenting for noncardiac surgery with VADs are becoming more frequent. Several considerations need to be observed in those supported by LVADs:

1) Adequate intravascular volume is needed at all times, as these devices are “preload” dependent and a decrease in left ventricle “preload” will diminish the pump flow. As such, anesthetic agents, fluid deficits (dehydration or bleeding), as well as the high intrathoracic pressure associated with general endotracheal intubation, may decrease venous return compromising hemodynamics. If the LVAD flow were to be higher than the available left ventricle preload, a suction event could occur where the walls of the ventricle collapse toward the inflow tract. This could either be asymptomatic or lead to decreased LVAD flows and right heart dysfunction with worsening hemodynamics.

2) Maintenance of afterload as LVADs are “afterload sensitive” and hypertensive response to inadequate anesthetic depth during laryngoscopy will precipitate a decrease in pump flow [2]. Similarly, hypotension should be avoided. A goal mean arterial pressure (MAP) between 70 and 90 mmHg is generally recommended, as there is a concern for end-organ hypoperfusion with MAPs < 70 mmHg [2].

3) Maintenance of heart rate and rhythm should be emphasized, as arrhythmias might lead to decreased pump flow.

4) Avoiding factors that could precipitate right-sided heart failure. Factors that increase pulmonary vascular resistance, such as variations in temperature, hypercarbia, acidosis, and hypoxia, should be avoided especially when the right ventricle is unsupported. Overzealous fluid resuscitation can lead to a decrease in left ventricular preload as right ventricular distention can limit forward stroke volume [2].

5) Careful airway manipulation when placing the endotracheal tube. Mucosal bleeding is a well-documented complication after LVAD implantation in the setting of telangiectasias/arteriovenous malformations (AVMs) formation. These vascular malformations are a significant cause of morbidity in patients with continuous flow (CF) LVADs, although the exact mechanism of AVM formation is still unclear. One of the proposed mechanisms is the decreased pulse pressure during continuous flow contributing to AVM development (diminished mucosal perfusion leads to regional hypoxia and new dysplastic vessel creation) [3]. A second factor contributing to bleeding could be the acquired von Willebrand syndrome commonly seen in patients with CF LVADs.

While general anesthesia is usually preferred due to proximity to the airway, thyroidectomies can be safely under various anesthetic techniques. Kim et al. recently described a case series of 18 patients undergoing thyroidectomy with target-controlled infusions of propofol and remifentanil in addition to lidocaine infiltration at the surgical site by the surgeon. While the authors report a positive experience with this trial in regards to no postoperative nausea and vomiting and no narcotic use in the recovery area, patients still experienced transient apneic and desaturation periods requiring jaw thrust as well as fluctuations in hemodynamics. It is also important to note that patients in this study were mainly healthy individuals - ASA class I and II [4].

Latifi et al. presented a case report of total thyroidectomy for a giant goiter during a medical mission [5]. Because the goiter compressed the trachea, the airway was unable to be secured, requiring the surgery to be performed with local anesthesia and ketamine for sedation. The patient tolerated the procedure well and there were no complications after five years.

Another described method is performing the thyroidectomy under a superficial or deep cervical plexus block only [6]. The block can be performed in conjunction with IV adjuncts such as midazolam or propofol. Ueshima et al. presented two patients who successfully underwent thyroid surgery with ultrasound-guided bilateral cervical plexus block and no sedation [7].

Spanknebel et al. reported a 1025 patient case series of thyroidectomies done with a combination of local anesthetic creating a local field and a superficial cervical plexus block and IV sedation using propofol [8]. It is important to note that, in this case series, a modified surgical technique was performed in light of using MAC as opposed to general anesthesia. This may not be an approach many surgeons are comfortable with. Also, almost 90% of the patients in the series were ASA classification I or II.

A small randomized study of 58 patients by Snyder et al. reported no difference in postoperative outcomes, complications, or patient satisfaction in those who underwent thyroid surgery with anterior field block and monitored anesthesia care using a combination of propofol, midazolam, and fentanyl as compared to those who underwent these procedures under general anesthesia [9]. The noted benefits in the local anesthesia group included a shorter patient stay in the recovery area as well as decreased healthcare costs.

For our patient, we chose a MAC technique/superficial cervical block in order to diminish the hemodynamic effects that could further impact LVAD physiology. Careful titration of the anesthetic agent (alfentanil) allowed us to avoid changes in preload and afterload seen with more commonly anesthetic agents and with induction and maintenance of general endotracheal anesthesia. In addition, since no airway intervention was necessary, the risk of airway bleeding complications was considerably reduced.

As to the choice of anesthetic agent, we chose an alfentanil infusion as the main sedating medication. Compared with the mainstay medications used during MAC (e.g. propofol), alfentanil is less likely to cause apnea and hypoventilation. Moreover, alfentanil has the advantage of having the quickest onset of all opioids and a short duration of action, making it easily titratable.

Various techniques have been used to provide adequate anesthesia for thyroidectomies. As anesthesiologists, we need to be familiar with different approaches, tailored specifically to the patient’s comorbidities. In our case, the presence of the LVAD physiology led us to choose an anesthetic technique with minimal hemodynamic consequences while maintaining optimal surgical conditions.

## Conclusions

Various techniques have been used to provide adequate anesthesia for thyroidectomies. As anesthesiologists, we need to be familiar with different approaches, tailored specifically to the patient’s comorbidities. In our case, the presence of the LVAD physiology led us to choose an anesthetic technique with minimal hemodynamic consequences while maintaining optimal surgical conditions.
